# Sociodemographic factors affecting not receiving COVID-19 vaccine in Japan among people who originally intended to vaccinate: a prospective cohort study

**DOI:** 10.3389/fpubh.2023.1290187

**Published:** 2023-12-06

**Authors:** Akiko Matsuyama, Takahiro Mori, Akira Ogami, Kosuke Mafune, Seiichiro Tateishi, Mami Kuwamura, Keiji Muramatsu, Yoshihisa Fujino, Koji Mori, CORoNaWork project

**Affiliations:** ^1^Department of Occupational Health Practice and Management, Institute of Industrial Ecological Sciences, University of Occupational and Environmental Health, Kitakyushu, Japan; ^2^Department of Work Systems and Health, Institute of Industrial Ecological Sciences, University of Occupational and Environmental Health, Kitakyushu, Japan; ^3^Department of Mental Health, Institute of Industrial Ecological Sciences, University of Occupational and Environmental Health, Kitakyushu, Japan; ^4^Disaster Occupational Health Center, Institute of Industrial Ecological Sciences, University of Occupational and Environmental Health, Kitakyushu, Japan; ^5^Department of Environmental Health, School of Medicine, University of Occupational and Environmental Health, Kitakyushu, Japan; ^6^Department of Public Health, School of Medicine, University of Occupational and Environmental Health, Kitakyushu, Japan; ^7^Department of Environmental Epidemiology, Institute of Industrial Ecological Sciences, University of Occupational and Environmental Health, Kitakyushu, Japan

**Keywords:** sociodemographic factors, COVID-19, side effects, financial impact, social support, Japan

## Abstract

**Objective:**

Vaccine hesitancy is a major issue for acquiring herd immunity. However, some individuals may go unvaccinated owing to inhibitory factors other than vaccine hesitancy. If there is even a small number of such people, support is needed for equitable vaccine distribution and acquiring herd immunity. We investigated sociodemographic factors that affected not undergoing COVID-19 vaccination in Japan among individuals who had strong intention to vaccinate before beginning the vaccination.

**Methods:**

We conducted this prospective cohort study on workers aged 20–65 years from December 2020 (baseline), to December 2021 using a self-administered questionnaire survey. There were 27,036 participants at baseline and 18,560 at follow-up. We included 6,955 participants who answered yes to this question at baseline: “Would you like to receive a COVID-19 vaccine as soon as it becomes available?” We applied multilevel logistic regression analyses to examine the association between sociodemographic factors and being unvaccinated at follow-up.

**Results:**

In all, 289 participants (4.2%) went unvaccinated. The odds ratios (ORs) for being unvaccinated were significantly higher for participants aged 30–39 and 40–49 than those aged 60–65 years. Being divorced, widowed, or single, having low income, and having COVID-19 infection experience also had higher ORs.

**Discussion:**

We found that some participants who initially had strong intention to vaccinate may have gone unvaccinated owing to vaccine side effects and the financial impact of absenteeism due to side effects. It is necessary to provide information repeatedly about the need for vaccination as well as social support to ensure that those who intend to vaccinate are able to do so when aiming for acquiring herd immunity through vaccination against COVID-19 as well as other potential infection pandemics in the future.

## Introduction

As with many viral infections, vaccination against COVID-19 is the most effective infection control (1). Various types of vaccines for COVID-19, including mRNA vaccines, have been developed over a short period of time and applied worldwide since December 2020 (2, 3). The efficacy and side effects differ according to the type of vaccine; however, many vaccines have been shown both to prevent onset and avert severity and death (4–6).

With an emerging epidemic like COVID-19, a high inoculation rate of effective vaccines can be used to establish herd immunity and control it (7). However, vaccine hesitancy—defined as a “delay in acceptance or refusal of vaccination despite availability of vaccination services”—hinders effective vaccination and poses a major public health issue (8). To reduce such hesitancy for COVID-19 in Japan, educational activities about the significance and risks of vaccination, free vaccination, and fair vaccine distribution began on February 17, 2021 for health-care workers; they were followed by older people and individuals with underlying medical conditions (9). Also, efforts have been made to create convenient vaccination sites and provide vaccines in the workplace (3). By December 2021, the second-dose vaccination among people aged over 12 years had been mostly completed, amounting to 73.4% of the total population (10).

Even though some people initially intended to be vaccinated, they later refuse vaccination owing to some inhibitory factors such as income factors —even though they too initially intended to receive it. If there is even a small number of such people, support is needed for equitable vaccine distribution and acquiring herd immunity. There is a necessity for a prospective cohort study or trajectory study to confirm whether people who initially intended to be vaccinated actually did so; however, few studies have examined the factors that influence vaccination decision. Sigerl et al. investigated COVID-19 vaccination in the United States; they found that 7% of people who were willing to be vaccinated in the baseline survey had become unwilling by the follow-up survey; however, the authors did not confirm actual vaccination rates and did not investigate the related factors (11).

As part of the Collaborative Online Research on Novel-coronavirus and Work (CORoNaWork) study (12), we asked participants about their intention to receive COVID-19 vaccination at baseline and their actual vaccination at follow-up. Using those data, we conducted a trajectory study to examine the sociodemographic factors that affected the actual vaccination rate of individuals who at baseline expressed strong intention to undergo vaccination.

## Methods

### Study design

We conducted this prospective cohort study as part of the CORoNaWork study. We carried out the baseline survey between December 22 to 25, 2020 and the follow-up survey between December 15 to 22, 2021, by which time the second-dose vaccination had been almost completed in Japan (10). Both surveys were conducted using self-administered questionnaires via the internet. Comprehensive details of the study protocol have been documented elsewhere (12). This study included participants aged 20 to 65 years who were employed at the time of the baseline survey (*n* = 33,087). Respondents were sampled taking into account region, occupation, and sex. After excluding 6,051 initial subjects who provided invalid responses, we finally included 27,036 in the database. The criteria of identifying responses as invalid were defined as follows: completion of the survey within a very short time (less than 6 min); body weight significantly below 30 kilograms; height considerably shorter than 140 centimeters; inconsistent responses to similar survey questions; and incorrect answers to a specific question designed to detect unreliable answers.

**Figure 1:**
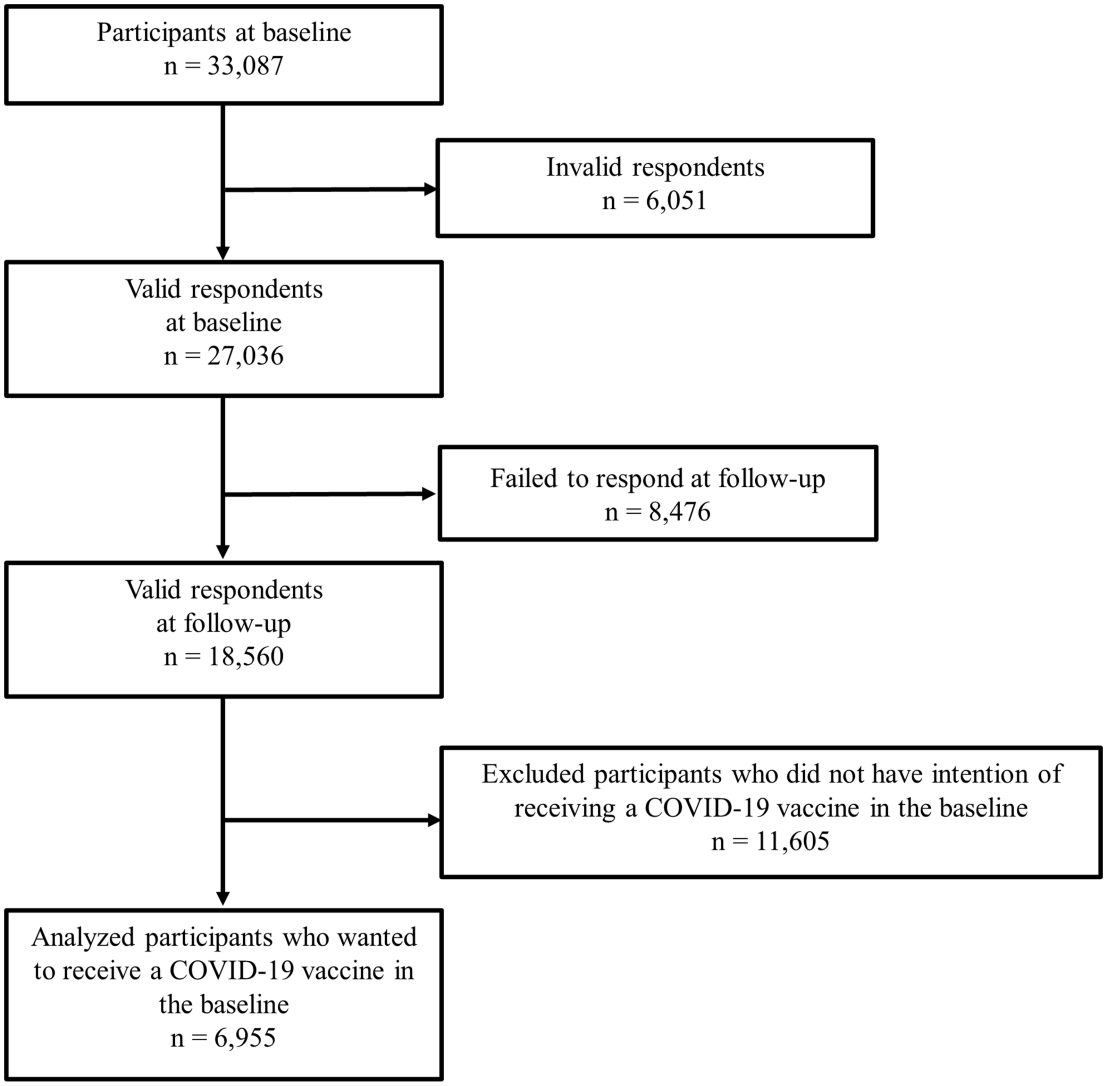
Flow diagram of this study.

In all, 18,560 participants (68.6%) undertook the follow-up survey. We conducted our analysis on 6,955 participants who answered yes to this question in the baseline survey, when it was unclear when the vaccination would begin in Japan: “Would you like to receive a COVID-19 vaccine as soon as it becomes available?” Figure 1 is a flow diagram for this study.

This study was approved by the Ethics Committee of the University of Occupational and Environmental Health, Japan (approval numbers: R2-079 and R3-006). Informed consent was obtained on the website from all participants.

### COVID-19 vaccination status

We asked participants this question in the follow-up survey: “What is your COVID-19 vaccination status?” Participants chose one of three options: vaccinated twice; vaccinated once; and unvaccinated. We created a binary variable by defining “unvaccinated” as not having received a COVID-19 vaccine and the other options as having the vaccine.

### Sociodemographic status

We investigated sex, age, marital status, annual household income, job type, and experience of COVID-19 infection. Age was classified into five groups: 20–29, 30–39, 40–49, 50–59, and 60–65 years. Marital status was classified into three categories: married, divorced or widowed, and single. Annual household income was classified into four groups: <4 million, 4–5.99 million, 6–8.99 million, and ≥ 9 million yen (US$1 equaled 109.75 yen in 2021) (13). Job type was classified into three categories: mainly desk work, jobs mainly involving interpersonal communication, and mainly physical work. For experience of COVID-19 infection, we asked this question at baseline: “Have you ever been infected with COVID-19?” Respondents answered yes or no. At follow-up, we asked this question: “Have you been diagnosed with COVID-19 since January 2021?” Likewise, respondents answered yes or no. We defined participants who answered yes to either question as having experienced COVID-19.

### Statistical analyses

We examined the association between sociodemographic factors and not receiving COVID-19 vaccination among respondents who had originally intended to do so. We estimated age-sex adjusted odds ratios (ORs) and multivariate-adjusted ORs using a multilevel logistic regression model nested in the prefecture of residence to consider regional differences in the infection status of COVID-19. The multivariate model was adjusted for sex, age, marital status, annual household income, job type, and experience of COVID-19 infection. We did not adjust for education because doing so would have been an over-adjustment. We also conducted a trend test with age and annual household income as continuous variables. A *p* value of less than 0.05 was considered statistically significant. We conducted all analyses using Stata statistical software (release 16; StataCorp LLC, College Station, TX, USA).

## Results

The characteristics of the participants appear in Table 1. Of the 6,955 participants analyzed, 4,382 were men (63.0%) and 2,573 women (37.0%). As of December 2021, 289 (4.2%) were unvaccinated.

**Table 1:** Caption.

	*N* (%)
Number of participants	6,955
Sex	
Men	4,382 (63.0%)
Age (years)	
20–29	276 (4.0%)
30–39	911 (13.1%)
40–49	1,871 (26.9%)
50–59	2,715 (39.0%)
60–65	1,182 (17.0%)
Annual household income (yen)	
<4 million	1,644 (23.6%)
4–5.99 million	1,602 (23.0%)
6–8.99 million	1,936 (27.8%)
≥9 million	1,773 (25.5%)
Marital status	
Married	4,302 (61.9%)
Divorced or widowed	696 (10.0%)
Single	1,957 (28.1%)
Job type	
Mainly desk work	3,492 (50.2%)
Mainly involving interpersonal communication	1,708 (24.6%)
Mainly physical labor	1,755 (25.2%)
Experience of COVID-19 infection	
Yes	152 (2.2%)
COVID-19 vaccination status	
Unvaccinated	289 (4.2%)

Table 2 shows the ORs for the association between sociodemographic factors and being unvaccinated among participants who originally intended to vaccinate. In the age-sex-adjusted analysis, compared with participants aged 60–65 years, ORs were significantly higher for those aged 20–29 (OR, 2.62), 30–39 (OR, 3.05), 40–49 (OR, 2.03), and 50–59 years (OR, 1.63). Divorced or widowed participants (OR, 2.72) and singles (OR, 2.43) had significantly higher ORs than married individuals. Regarding annual household income, compared with ≥9 million yen, participants with <4 million (OR, 2.46) and 4–5.99 million yen (OR, 1.70) had significantly higher ORs. Mainly physical workers had a higher OR than mainly desk workers (OR, 1.49). Participants with experience of COVID-19 infection had a higher OR than those without experience (OR, 1.85). In the multivariate-adjusted analysis, participants aged 30–39 (OR, 2.46) and 40–49 years (OR, 1.70), divorced or widowed people (OR, 2.25) and singles (OR, 2.04), participants with an income of <4 million yen (OR 1.63), and those with experience of COVID-19 infection (OR, 2.02) had still significantly higher ORs. We also observed a linear relationship between being unvaccinated and age (*P* for trend = 0.009) and income (*P* for trend = 0.028).

**Table 2:** Association between sociodemographic factors and going unvaccinated among participants who originally intended to vaccinate.

	Unvaccinated rate	Age-sex adjusted			Multivariate adjusted*		
	%	OR	95% CI	*p* value	OR	95% CI	*p* value
Sex							
Men	3.8	1.00			1.00		
Women	4.8	1.01	0.78–1.31	0.953	0.84	0.64–1.10	0.199
Age (years)				<0.001†			0.009†
20–29	5.8	2.62	1.35–5.09	0.004	1.88	0.95–3.74	0.071
30–39	6.7	3.05	1.88–4.95	0.000	2.46	1.49–4.04	0.000
40–49	4.5	2.03	1.30–3.18	0.002	1.70	1.08–2.69	0.022
50–59	3.7	1.63	1.06–2.51	0.026	1.51	0.98–2.34	0.064
60–65	2.3	1.00			1.00		
Annual household income (yen)				<0.001†			0.028†
<4 million	6.5	2.46	1.72–3.52	<0.001	1.63	1.11–2.40	0.013
4–5.99 million	4.6	1.70	1.17–2.48	0.006	1.33	0.90–1.96	0.151
6–8.99 million	3.2	1.14	0.77–1.69	0.503	1.04	0.70–1.53	0.858
≥9 million	2.7	1.00			1.00		
Marital status							
Married	2.6	1.00			1.00		
Divorced or widowed	6.5	2.72	1.88–3.92	<0.001	2.25	1.53–3.31	<0.001
Single	6.8	2.43	1.84–3.20	<0.001	2.04	1.52–2.74	<0.001
Job type							
Mainly desk work	3.7	1.00			1.00		
Mainly involving interpersonal communication	3.6	0.92	0.68–1.26	0.612	0.87	0.64–1.20	0.402
Mainly physical labor	5.6	1.49	1.13–1.95	0.004	1.29	0.98–1.71	0.073
Experience of COVID-19 infection							
No	4.1	1.00			1.00		
Yes	7.9	1.85	1.01–3.40	0.046	2.02	1.09–3.72	0.025

*Adjusted for sex, age, marital status, annual household income, job type, and experience of COVID-19 infection.

† *P* for trend.

All analyses used multilevel logistic regression nested in the prefecture of residence.

OR, odds ratio; CI, confidence interval.

## Discussion

We conducted a trajectory study about COVID-19 vaccination; we examined the sociodemographic factors related to vaccination among participants who at baseline had strong intention to do so. We found that young to middle-aged participants, being divorced, widowed, or single, low-income earners, and individuals with experience of COVID-19 infection were more likely to be unvaccinated. We assumed they became hesitant about vaccination owing to perceived risks associated with vaccine side effects or being unable to receive vaccination for financial reasons.

The reason for becoming hesitant about undergoing vaccination was changing in their risk perceptions related to the vaccines owing to acquiring various information over time. Vaccination decisions are generally influenced by perceptions of two risks: the risk of not being vaccinated (e.g., risk of becoming infected or infection becoming severe); and the risk of vaccination (e.g., vaccine side effects) (14). COVID-19 vaccines were manufactured shortly after the pandemic was declared; that raised overall concerns about side effects and future safety (15). In Japan, two mRNA vaccines, one from Pfizer Inc. and one from Moderna Inc., were used and reportedly caused both local symptoms and systemic symptoms as side effects; those side effects occurred more frequently than with seasonal influenza vaccines (16–19). Thus, we believe those side effects and concerns about vaccine safety owing to the short production time affected the participants’ intentions to vaccinate.

Another factor is that employees may have been obliged to be absent from work owing to vaccination side effects and thus went unvaccinated. In Japan, efforts were made to decrease vaccine hesitancy through access issues by ensuring free vaccination, fair vaccine distribution, and convenient vaccination sites (such as workplace vaccination) (3, 9). However, compensation for absenteeism on the day of vaccination and through side effects was determined by companies (3, 20). Therefore, participants who were concerned about vaccination side effects and who were unable or unwilling to take time off work for financial or other reasons may have been less likely to be vaccinated. On the basis of the above two factors, we now discuss each of the study covariates.

We found that younger or middle-aged participants were less likely to be vaccinated than older ones. Younger or middle-aged people were reportedly less likely to be severely infected with COVID-19 than older individuals, which may have reduced infection risk perceptions among the former (21). The Moderna vaccine had a higher rate of systemic side effects, and especially among young men, a higher incidence of myocarditis and pericarditis compared to the Pfizer vaccine (16, 22); however, in Japan, the Pfizer vaccine was mainly used in clinics, whereas the Moderna vaccine was applied in mass vaccination by local governments and in occupational vaccination (23). Accordingly, we observed a higher proportion of young to middle-aged participants who had received the Moderna vaccine. Thus, young to middle-aged participants may have believed that the risk of getting vaccinated outweighed the risks of not doing so.

Among low-income earners, we found that many went unvaccinated even though initially they had intended to be vaccinated. Low-income earners have a high unemployment rate; even during COVID-19, sickness presenteeism was reportedly high (24, 25). It was evidently difficult for such people to take time off work for vaccination. If they did not receive leave compensation for vaccination or its side effects, they may have refused vaccination through concerns about the direct impact on their incomes owing to taking time off work and even about their employment status as a result of absenteeism. Additionally, individuals who were single, divorced, or widowed, as well as manual workers, were more likely to be unvaccinated, possibly because of their lower incomes (26–28). In particular, manual workers are mainly occupied on-site, making it challenging for them to take time off from work to maintain stable production (25).

With respect to participants with experience of COVID-19 infection going unvaccinated, decreased awareness of the need for vaccination and increased concern about vaccine side effects both possibly had an impact. COVID-19 vaccination is recommended regardless of infection experience, but the reinfection rate reportedly decreases for a while after infection owing to the production of neutralizing antibodies (29, 30). Further, individuals who have been infected are more likely to have vaccine side effects, which may increase their concerns: they may have slowly acquired this information and chose not to receive vaccination (31, 32). Others may have missed the opportunity to be vaccinated because they were infected with COVID-19 shortly beforehand—even though they originally planned to be vaccinated.

We found that a small proportion (4%) of respondents did not receive COVID-19 vaccination despite a strong intention to do so. Sociodemographic factors (such as being young to middle-aged; being single, divorced, or widowed; low-income earners; manual workers; and individuals with experience of COVID-19 infection) may have influenced the vaccination decision. Attribute differences in concerns about vaccine side effects and the impact of side effects on work may also have played a role. Our findings offer suggestions to consider when aiming for acquiring herd immunity through vaccination in future epidemics. First, the national government has to consider distributing vaccinations by age according to reports of vaccine side effects and consider the type and location of vaccines being more freely selected. Second, it is necessary to explain repeatedly the benefits and needs for vaccination and maintain or enhance the intention to vaccinate. For individuals with infection experience, it is important to provide appropriate information about the following: antibody production due to infection being low compared with the amount of antibody produced by vaccination; the possibility of reinfection; and vaccination being important even after infection owing to the effects of new mutant strains of SARS-CoV-2 (33, 34). Third, it is necessary to consider introducing a direct compensation system for workers and a support system for companies so that employees can take leave owing to vaccine side effects.

This study has several limitations. First, we investigated the reasons for participants going unvaccinated despite initially strongly intending to vaccinate; however, the number of people targeted was small, which may have affected the analysis results. Second, the survey was conducted via the Internet, so there is a limit to its generalizability. Notably, some socially vulnerable groups may have been unable to participate because they lacked Internet access for financial reasons. Third, we did not investigate the exact reasons for going unvaccinated, which we consider to be the most significant limitation of our study. Further research in this regard is required. Fourth, we used responses about infection experience up to December 2021, but we also included in our analysis participants with COVID-19 infection after vaccination; thus, our results may be an underestimate.

In conclusion, we observed that sociodemographic factors (such as being young to middle-aged; being divorced, widowed, or single; having low income; and having COVID-19 infection experience) affect going unvaccinated even if there was initially strong intention to do so. Concerns about vaccine side effects and the impact of side effects on work may have played a role in the vaccination decision. It is necessary to explain repeatedly the need for vaccination and to provide social support to ensure that individuals who intend to vaccinate are able to do so when aiming for acquiring herd immunity through vaccination against COVID-19 as well as other potential infection pandemics in the future.

## Data availability statement

The raw data supporting the conclusions of this article will be made available by the authors, without undue reservation.

## Ethics statement

The studies involving humans were approved by the Ethics Committee of the University of Occupational and Environmental Health, Japan (approval numbers: R2-079 and R3-006). The studies were conducted in accordance with the local legislation and institutional requirements. The participants provided their written informed consent to participate in this study.

## Author contributions

AM: Formal analysis, Methodology, Writing – original draft. TM: Formal analysis, Methodology, Writing – review & editing. AO: Data curation, Funding acquisition, Investigation, Writing – review & editing. KMa: Data curation, Investigation, Writing – review & editing. ST: Data curation, Funding acquisition, Investigation, Writing – review & editing. MK: Data curation, Investigation, Writing – review & editing. KMu: Data curation, Funding acquisition, Investigation, Writing – review & editing. YF: Data curation, Funding acquisition, Investigation, Project administration, Supervision, Writing – review & editing. KMo: Conceptualization, Data curation, Funding acquisition, Investigation, Methodology, Supervision, Writing – review & editing.
